# Asynchronous Bilateral Ovarian Torsion: Three Cases, Three Lessons

**DOI:** 10.1155/2017/6145467

**Published:** 2017-12-18

**Authors:** M. C. Lucchetti, C. Orazi, A. Lais, M. L. Capitanucci, P. Caione, H. Bakhsh

**Affiliations:** ^1^Nephro-Urology Department, Bambino Gesù Children's Hospital, Rome, Italy; ^2^Imaging Department, Bambino Gesù Children's Hospital, Rome, Italy; ^3^College of Medicine, Clinical Science Department, Princess Nourah Bint Abdulrahman University, Riyadh, Saudi Arabia

## Abstract

**Background:**

Ovarian torsion (OT) is a serious condition, and delay in surgical intervention may result in loss of the ovary. Children and adolescents who have suffered from ovarian torsion may be at risk for asynchronous torsion of the contralateral ovary.

**Study objective:**

Three cases of asynchronous bilateral ovarian torsion were reported to analyse clinical history of three patients, to review the current literature, and to draw a conclusion for future treatment.

**Design:**

Case reports and review of the literature.

**Result:**

When a prepubertal girl presents with an ovarian torsion, several considerations have to be taken in account in order to preserve her future fertility; in particular, the pediatric surgeon/gynecologist has to preserve as much as possible the twisted ovary in addition to considering the fate of the contralateral ovary.

**Summary and Conclusions:**

Pelvic pain in a young girl has always raised the clinical suspect of an ovarian torsion; the possibility of asynchronous bilateral ovarian torsion is rare, but it is described in the literature and has catastrophic consequences; this condition has to be known and treated in the proper way by pediatric surgeons as well as by gynecologists in order to maximize the future fertility of the young patients.

## 1. Introduction

Ovarian torsion (OT) is defined as partial or complete torsion of the ovarian vascular pedicle producing cessation of circulation that is initially venous and lymphatic and consequently becomes arterial occlusion which may occur as a resultant of edema progression [1].

Asynchronous bilateral ovarian torsion (ABOT) is defined as torsion of each ovary at different settings [2].

Complete occlusion of the ovarian blood supply will ultimately result in loss of ovarian function and necrosis of the torsed tissues, and life-threatening complications such as hemorrhage or peritonitis could occur as additional potential adverse effects [3].

When OT occurs, the ovary typically rotates around both the infundibulopelvic ligament and the utero-ovarian ligament. The fallopian tube often twists along with the ovary; this is referred to as adnexal torsion.

The incidence of OT in pediatric population is between 4.9 and 20 in 100,000 [4, 5]. There are some data regarding the rate of torsion among patients presenting to gynecologic care as an acute care setting, and ovarian torsion accounted for 2.7% of emergency surgeries [6].

In children under the age of 15 years, normal ovaries have been demonstrated in over 50% of patients with ovarian torsion [7].

ABOT of normal ovaries has also been reported [8] as summarized in Table 1 [2, 9–17].

Torsion usually occurs infrequently in premenarchal girls. However, when an ovarian mass is present, torsion is a common complication.

Torsion accounts for 20 to 30% of ovarian surgeries in the pediatric group population [18].

Rotation of the infundibulopelvic ligament causes compression of the ovarian vessels and impedes lymphatic and venous outflow and arterial inflow. However, the arterial supply to the ovary is not initially interrupted to the same degree as the venous drainage since the muscular arteries are less compressible than the thin walls of the veins. Continued arterial perfusion in the setting of blocked outflow leads to ovarian edema with marked ovarian enlargement and further vascular compression resulting in ovarian ischemia, necrosis, infarction, and local hemorrhage.

However, hemorrhage requiring blood transfusion or sepsis has rarely been reported [19].

The necrotic tissue will involute over time, and if pelvic adhesion formed, this could result in pelvic pain or infertility.

Mechanism of torsion of normal ovaries in the absence of cysts or masses is unclear. This has been found in patients of all ages, particularly in premenarchal girls. The utero-ovarian ligament is normally elongated in premenarchal girls and then shortens as they mature through puberty.

Hypermobility due to an elongated utero-ovarian ligament and hyperlaxity of mesosalpinx or mesoovarium may be contributing factors [7].

In addition, impeded venous return with stasis and congestion results in a heavier ovary [20].

OT is a gynecological emergency that requires prompt surgical intervention, but it can be difficult to distinguish clinically from appendicitis and other causes of acute abdominal pain [1].

The classic presentation of ovarian torsion is the acute onset of moderate to severe pelvic pain, often with nausea and vomiting [3].

However, the presentation may vary, and many symptoms and signs that accompany torsion are also associated with other conditions. Fever may be a marker of adnexal necrosis, particularly in the setting of leukocytosis.

Findings on physical examination are variable. Most patients exhibit pelvic and/or abdominal tenderness, although tenderness on examination is absent in as many as one-third of the patients [19].

The pattern of pain associated with ovarian torsion is variable, and thus, the differential diagnosis also includes other conditions that are associated with acute or chronic pelvic pain.

Appendicitis is another etiology of pelvic pain, nausea, and fever that may be difficult to differentiate from adnexal torsion. Currently, these two conditions are differentiated by the patient's symptoms, physical examination to localize the pain, and by the presence of characteristic imaging findings [21].

Infants with ovarian torsion present with feeding intolerance, vomiting, abdominal distension, and fussiness/irritability [3].

Prompt diagnosis is important to preserve ovarian and/or tubal function and to prevent other associated morbidities. However, making the diagnosis can be challenging because the symptoms are relatively nonspecific [22].

The clinical diagnosis of adnexal torsion in children is often uncertain, and delay in surgical intervention frequently may cause the necrosis of adnexal structures necessitating resection [23].

A definitive diagnosis of ovarian torsion is made by direct visualization of a rotated ovary at the time of surgical evaluation [22].

Once a girl has lost one ovary, there is a risk of ABOT, which may result in catastrophic sequelae [9].

Three illustrative cases with asynchronous bilateral adnexal torsion in prepubertal girls are presented in this article.

## 2. Case Reports

### 2.1. Case 1

A girl of 3 years and 4 months of age was admitted to our department because of acute low abdominal pain, lasting more than 48 hours. At 14 days of life, she was taken to the operating room (OR), and a right oophorectomy was performed based on a prenatal diagnosis of right ovarian torsion secondary to ovarian cyst around 8 cm in size. Pelvic ultrasound and MRI showed a left paraovarian mass with a suspicion of torsion. An emergency laparotomy was performed. Intraoperative finding showed that the left ovary with a black-bluish color was torsed twice, and a slight revascularization after detorsion was noted. Ovarian biopsy was performed. The ovary was preserved, and an oophoropexy was done. The decision to preserve such a devascularized ovary was made to delay the decision to remove the remaining ovary. Pathology was positive for a massive hemorrhagic infarction of the ovarian cortex.

Regular follow-up pelvic ultrasound showed a normally looking left ovary, and color-doppler demonstrated a normal blood flow.

### 2.2. Case 2

A 9-year and 1-month age premenarchal girl was evaluated in the emergency department because of lower abdominal pain and vomiting. Pelvic ultrasound showed enlarged left ovary. Past surgical history was detorsion of the right ovary and oophoropexy in retrouterine position 4 months earlier.

Normal right ovary and left ovarian torsion were found intraoperatively; detorsion of the left ovarian tissues and biopsy were performed, and left oophoropexy was not done.

Left ovarian cortex with hemorrhagic infarction was confirmed by histopathology.

On regular follow-up, physical examination showed that Tanner's stage was (P4, B4), and pelvic ultrasound showed normal ovaries with small, diffuse follicles with normal uterus size for her age (6.1 cm in length and 4.5 cm in width) and thin endometrial thickness.

### 2.3. Case 3

A 9-year-old girl was admitted complaining of abdominal pain in the right iliac fossa. US scan revealed fluid-filled material and retrouterine mass of 50–60 mm. At surgical exploration, complete torsion of the right ovary was found. The ovary was completely destroyed by hemorrhagic infarction. The left ovary and fallopian tube were normal. Right salpingo-oophorectomy was performed. The girl recovered uneventfully.

Subsequent follow-up US scans taken yearly were all normal.

Three years later, she was readmitted to the hospital with acute lower left abdominal pain. Her first menstrual cycle was 3 weeks prior to this presentation. Pelvic US scan showed that the left ovary was enlarged (60 mm), hypoechogenic, and filled with fluid material. She was taken to the OR, and torsion of the left ovary was found. Detorsion and warmth application reestablished good blood supply within minutes. The left ovary was fixed to the posterior abdominal wall with absorbable sutures.

A follow-up ultrasound at the age of 13 was unremarkable.

One year later, the patient presents to the hospital with acute lower abdominal pain (RIF). Doppler pelvic ultrasound and MRI were performed which showed an enlarged left ovary (67 mm) with follicles seen at periphery with complete absence of arterial supply. Intraoperative findings showed that a complete left ovarian torsion was found, no sign of the previous oophoropexy could be seen, detorsion of the left ovary was done, and then, the blood supply was restored promptly. The left ovary was refixed to the posterior abdominal wall using nonabsorbable sutures.

She was recovered uneventfully.

Regular follow-up with our pediatric gynecologist was performed periodically.

### 2.4. First Lesson (First Case)

Even ovaries with bad appearance and poor histology have to be detorsed and conservatively treated.

### 2.5. Second Lesson (Second Case)

When an ovarian torsion happens without an underlying ovarian mass or cyst in a premenarchal girl, at least the detorsed gonad has to be fixed. It is a matter of debate whether the contralateral ovary has to be pexed as well.

### 2.6. Third Lesson (Third Case)

When a pexy is needed, the surgical technique may be different, but the suture has to be nonabsorbable.

## 3. Discussion

Ovarian torsion (OT) is a surgical emergency because of the potential for reproductive and hormonal compromise [1].

The right ovary appears to be more likely torsed than the left, possibly because the right utero-ovarian ligament is longer than the left and/or that the presence of the sigmoid colon in the left side of the colon may help to prevent torsion [6, 24].

The primary risk factor for ovarian torsion is an ovarian mass, particularly a mass that is 5 cm in diameter or larger [25].

It is important to note that torsion may occur in the presence of normal ovaries, particularly in the pediatric population [18].

The recurrence risk of OT varies with the etiology of the initial event, and about 11% of the patients have normal ovaries [6, 19].

After a review of the English-language literature, we were able to document 29 ABOT cases, recurrence, and surgical interventions as summarized in Table 2 [1, 2, 8, 9, 12, 15, 16, 26–40].

Pelvic ultrasound is the first-line imaging study for patients with suspected ovarian torsion. Pelvic magnetic resonance imaging (MRI) or computed tomography (CT) scan is not usually ordered for the evaluation for adnexal torsion.

Ultrasound is less expensive than CT and MRI, and it has similar diagnostic performance.

MRI and CT may be helpful if findings on ultrasound are equivocal [41].

The decision to proceed with a surgical evaluation is based upon a clinical diagnosis of ovarian torsion.

The goals of the intraoperative evaluation are to confirm the presence of torsion and evaluate the viability of the ovary and tube. Most torsed ovaries are considered potentially viable, unless there is a clearly necrotic appearance.

The standard approach to determining the viability of a torsed ovary is gross visual inspection. An ovary that is dark and enlarged likely has vascular and lymphatic congestion and may have hemorrhagic lesions. Commonly, ovaries with this appearance have been thought to be nonviable. However, multiple studies have found that many women (even those with an ovary, that is, blue or black) retain ovarian function following detorsion [42, 43].

In studies with ultrasound follow-up, the rate of follicular development after detorsion was 80% or higher [43].

The mainstay of treatment of ovarian torsion is swift operative evaluation to preserve ovarian function and prevent other adverse effects (such as hemorrhage, peritonitis, and adhesion formation).

Oophorectomy should be reserved for necrotic/gelatinous/dead tissue.

It appears that detorsion is associated with continued ovarian function in many patients [42].

The key factor is to perform detorsion as quickly as possible [5].

There is also no evidence of an increase of adverse events with detorsion. There was no increase in postoperative complications in those who underwent detorsion with cystectomy compared with salpingo-oophorectomy [44].

Detorsion consists of untwisting the torsed ovary and any other torsed structure.

While the benefits of conservative surgery appear to outweigh the theoretical surgical risks of detorsion, irreversible ischemic damage to the adnexa can occur and may lead to infection if a necrotic ovary is retained. Postoperative care and instructions following detorsion should include observation for signs of peritonitis or sepsis (like fever, abdominal pain, peritoneal signs, and hemodynamic instability) [45].

Patients with an ovary that is apparently necrotic (black color combined with loss of normal anatomic structure and a diminished size) during intraoperative evaluation should undergo salpingo-oophorectomy.

Oophoropexy can be performed in children with ovarian torsion who do not have an ovarian mass, but not in those with an ovarian mass present at the time of torsion. Oophoropexy can also be performed in girls and young women who have previously undergone an oophorectomy for prior ovarian torsion. The procedure can be performed laparoscopically and typically shorten the utero-ovarian ligament, or if the ovary is greatly enlarged without a discrete mass, then it can be sutured to the uterosacral ligament [46].

Oophoropexy has been proposed as a means of decreasing future reproductive harm by decreasing the risk of recurrent OT [47].

The exact role of oophoropexy remains unclear. Some have proposed a theoretical negative effect of oophoropexy on future fertility because of alteration in anatomy [4], and not surprisingly, some authors have discouraged its routine use [48].

Oophoropexy does not guarantee that a future torsion will be prevented because recurrence of OT after oophoropexy has been documented [17].

## 4. Conclusions

Conservative treatment of ovarian torsion (with or without ovarian pathology predisposing to torsion) is mandatory, particularly in the pediatric age group, because ABOT is a rare but potentially catastrophic event.

Pelvic ultrasound has to be performed without delay in any girl with previous ovarian torsion presenting with acute lower abdominal pain.

Contralateral pexy should be considered in all cases of ovarian torsion, even when the treatment has been conservative and the torsed ovary itself has been fixed.

Oophoropexy has to be realized with permanent suture because an absorbable pexy may completely disappear without any residual scar.

## Figures and Tables

**Table 1:** The reported cases of ABOT in the literature.

Authors	Diagnosis
Sutton [10]	1st description of adnexal torsion
Warnek [11]	1st reported case of bilateral adnexal torsion
Baron [12]	1st description of asynchronous bilateral adnexal torsion in childhood
Eckler et al. [13]	16 cases reported of bilateral torsion
Ozcan et al. [14]	17 cases reported of asynchronous bilateral adnexal torsion
Beaunoyer et al. [2]	4 cases reported (described) of asynchronous bilateral adnexal torsion
Varras et al. [15]	1 case reported of asynchronous bilateral adnexal torsion
Svensson et al. [16]	1 case reported of asynchronous bilateral ovarian torsion
Fuchs et al. [17]	4 cases reported of adnexal torsion
Kurtoglu et al. [9]	1 case reported of asynchronous bilateral ovarian torsion
Current study	3 cases of asynchronous bilateral ovarian torsion

**Table 2:** Reported cases of ABOT in the premenarchal age group.

Case no. and year	Age at time of first torsion (yr)	Age at time of second torsion (yr)	Interval between surgery	Affected side/surgical procedure at time of first torsion	Affected side/surgical procedure at time of second torsion	Castration
(1 case) 1934 [12]	7	9	2 years and 3 months	R/SOP	L/SOP	Yes
(1 case) 1980 [26]	12	12	6 weeks	R/SOP	L/SOP	Yes
(1 case) 1981 [27]	3	6	3 years	R/SOP	L/SOP	Yes
(1 case) 1984 [28]	7	8	2 years	R/SOP	L/SOP	Yes
(1 case) 1986 [29]	6	8	2 years	L/SOP	R/detorsion	No
(1 case) 1987 [30]	7	9	2 years	R/SOP	L/SOP	Yes
(1 case) 1989 [31]	6.5	10.5	4 years	L/SOP	R/SOP	Yes
(1 case) 1990 [32]	3.5	10.5	7 years	R/SOP	L/SOP	Yes
(1 case) 1990 [8]	8.5	9.5	1 year	R/SOP	L/SOP	Yes
(1 case) 1993 [33]	10	11	8 months	R/SOP	L/detorsion + OPXY	No
(1 case) 1996 [34]	10	12	2 years	L/SOP	R/detorsion + PLICA	No
(1 case) 1997 [35]	UK	13	UK	L/INCD	R/detorsion + PLICA	No
(1 case) 2000 [36]	8	17	9 years	R/SOP	L/detorsion + OPXY	No
(1 case) 2000 [37]	4.5	6	17 months	L/SOP	R/detorsion + OPXY	No
(1 case) 2000 [38]	UK	9	UK	L/INCD	R/detorsion	No
(1 case) 2002 [39]	9	10	7 months	R/SOP	L/detorsion + OPXY	No
(1 case) 2002 [39]	12	12	5 months	R/SOP	L/detorsion + OPXY	No
(4 cases) 2004 [2]	Mean age 10.6 years (ranging from 3.3 months to 13.1 years)	—	Range between 7 and 30 months	For all 4 cases: L/SOP	For all 4 cases: R/detorsion + OPXY	Yes
(1 case) 2005 [15]	13	13	20 days	R/SOP	L/SOP	Yes
(1 case) 2006 [1]	11	12	1 year	Right oophorectomy	L/detorsion + OPXY	Yes
(1 case) 2008 [16]	6	6	1 year	R/SOP	L/detorsion + OPXY	Yes
(1 case) 2013 [40]	8	8	4 months	R/detorsion	Underwent surgical exploration 4 times, intraoperative procedure was performed: L/detorsion + OPXY and bilateral shortening of ovarian ligaments	No
(1 case) 2014 [9]	9	12	1 month	R/SOP	L/SOP	Yes
Current case	14 days of her life	3 years and 4 months	3 years	R/SOP secondary to ovarian cyst	L/detorsion + OPXY	Yes
Current case	8 years and 8 months	9 years and 1 month	4 months	R/detorsion + OPXY	L/detorsion	No
Current case	9	12	3 years	R/SOP	Underwent surgical exploration 2 times, intraoperative procedure was performed: L/detorsion + OPXY	Yes

R: right; SOP: salpingo-oophorectomy; L: left; OPXY: oophoropexy; PLICA: plication of the utero-ovarian ligament; UK: unknown; INCD: incidentally found.
